# Correlations of CT scan with high-sensitivity C-reactive protein and D-dimer in patients with coronavirus disease 2019

**DOI:** 10.12669/pjms.36.6.2961

**Published:** 2020

**Authors:** Jin Zhu, Cheng Chen, Rongshu Shi, Bangguo Li

**Affiliations:** 1 Jin Zhu, Department of Intervention, Affiliated Hospital of Zunyi Medical University, Zunyi 563003, P.R. China; 2 Cheng Chen, Department of Thoracic Surgery, Affiliated Hospital of Zunyi Medical University, Zunyi 563003, P.R. China; 3 Rongshu Shi, Department of Intervention, Affiliated Hospital of Zunyi Medical University, Zunyi 563003, P.R. China; 4 Bangguo Li, Department of Imaging, Affiliated Hospital of Zunyi Medical University, Zunyi 563003, P. R. China

**Keywords:** coronavirus disease 2019, pulmonary fibrosis, D-dimer, high-sensitivity C-reactive protein

## Abstract

**Objectives:**

To study the correlations of CT scan with high-sensitivity C-reactive protein (hs-CRP) and D-dimer in patients with coronavirus disease 2019 (COVID-2019).

**Methods:**

From January to March 2020, COVID-19 patients were divided into two groups according to the Diagnosis and Treatment Protocol for Novel Coronavirus Pneumonia (trial version 7), with mild and ordinary cases as Group-1 and critical and severe cases as Group-2. The chest CT scan results, hs-CRP, D-dimmer levels of the two groups from admission to discharge were compared by the χ^2^ test or Fisher’s exact test. The quantitative data were represented as mean ± standard deviation ( x ± s). Intergroup comparisons were performed by the independent samples t test, and the ineligible data were subjected to the nonparametric rank sum test. Binary logistic regression model was used for multivariate correlation analysis, using independent variables that were significant in univariate analysis. The correlations between the above indices were analyzed.

**Results:**

In Group-1, there were two cases of normal chest CT scan results, one case of fibrosis, and 25 cases of abnormalities during the first diagnosis, mainly manifested as single or scattered ground-glass shadows. After treatment, the CT scan results became normal. The chest CT scan of Group-2 showed abnormalities, including 21 cases of multiple ground-glass shadows, and six cases of multiple consolidations accompanied by ground-glass shadows, who were critically ill and died. In addition, there were 16 cases of multiple ground glass shadows with partial consolidation, and the CRP and D-dimer levels of Group-2 were significantly higher than those of Group-1. Chest CT scan results were significantly positively correlated with CRP and D-dimer levels (P<0.05).

**Conclusion:**

The chest CT scan results of COVID-19 patients are characteristic, being correlated with CRP and D-dimer levels. D-dimer and CRP levels significantly increase in most severe and critical patients, which are closely related to their prognosis. The indices may play predictive roles in clinical treatment and prognosis evaluation.

## INTRODUCTION

In December 2019, patients with Coronavirus Disease 2019 (COVID-19) were first identified and diagnosed in Wuhan, Hubei province.^1^ The International Committee on Taxonomy of Viruses officially named the virus that caused the disease as severe acute respiratory syndrome coronavirus 2 on February 10th, 2020.^2^,^3^ The main clinical manifestations of COVID-19 patients mainly included fever, dry cough and fatigue, and the diagnosis was mainly based on the positivity of coronavirus nucleic acid detected by reverse transcription-polymerase chain reaction (RT-PCR) for the respiratory samples of suspected patients with epidemiological history and corresponding symptoms.^1^

CT scores and serum high-sensitivity C-reactive protein (hs-CRP) level have good consistency, and the combination of them can dynamically and effectively assess disease progression and therapeutic effects.^4^ Hs-CRP is an inflammatory response protein, the increase of which in serum can reflect the activation of inflammatory response.^5^ In patients with thrombosis, the increase in its serum level initiates the coagulation cascade reaction, and accelerates the formation of thrombi by activating the complement system and initiating the exogenous coagulation pathway.^6^ The systemic anatomy of a dead case of COVID-19 showed that there was microthrombosis in the pulmonary artery.^7^ The thrombosis of pulmonary arterioles in patients with severe COVID-19 can aggravate the progression of pulmonary lesions or delay the absorption of lesions.

In patients recovering from COVID-19 (without severe respiratory distress during the disease course), the lung abnormalities disclosed by chest CT scan became most severe about 10 days after the initial symptoms appeared.^8^ In this study, the correlations of chest CT scan results with hs-CRP and D-dimer levels in 71 mild and severe cases upon the first diagnosis were analyzed. The severity of chest CT lesions was positively correlated with hs-CRP and D-dimer levels which significantly increased in the patients with severe COVID-19. According to the pharmaceutical monitoring of the prevention and treatment for COVID-19-related circulatory system thrombosis,^9^ the clinical monitoring of the above indices can evaluate the prognosis and therapeutic efficacy.

Seventy-one COVID-19 patients diagnosed in Ezhou Central Hospital during the medical aid to Hubei province from January 3rd to February 26th, 2020 were retrospectively analyzed, all of whom were hospitalized after being detected positive by RT-PCR. According to the Diagnosis and Treatment Protocol for Novel Coronavirus Pneumonia (trial version 7),^1^ the patients with COVID-19 were divided into two groups, with mild and ordinary cases as Group-1 (n=28) and critical and severe cases as Group-2 (n=43). In Group-1, there were 18 males and 10 females aged 9-84 years old, with an average of (15.971 ± 49.607). In Group-2, there were 26 males and 17 females aged 35-94 years, with an average of (16.674 ± 60.884). The chest CT foci as well as changes of hs-CRP and D-dimer levels from admission to discharge were recorded.

With the patient breathing calmly, CT scan was conducted from the entrance of the thorax to the lower part of the diaphragm. Scan parameters: Tube voltage, 120 kV; tube current, 320 mA; scan pitch, 0.875; slice thickness, 5 mm; slice spacing, 5 mm; matrix, 512 × 512. All CT images were obtained through lung window and mediastinal window.

In the early morning, 3-5 ml of fasting venous blood was drawn from each patient, and the serum was collected after centrifugation to measure hs-CRP and D-dimer levels using an automatic biochemical analyzer. Blood collection and CT scan were carried out on the same day.

All data were statistically analyzed by SPSS 21.0 software. The numerical data were expressed as case number (%). Intergroup comparisons were performed by the χ^2^ test or Fisher’s exact test. The quantitative data were represented as mean ± standard deviation ( x ± s). Intergroup comparisons were conducted by the independent samples t test, and the ineligible data were subjected to the nonparametric rank sum test. Binary logistic regression model was used for multivariate correlation analysis, using independent variables that were significant in univariate analysis. The results were expressed as corrected odds ratio (OR) and corresponding 95% confidential interval (CI). P<0.05 was considered statistically significant.

## RESULTS

### CT scan results

**Fig. 1:**
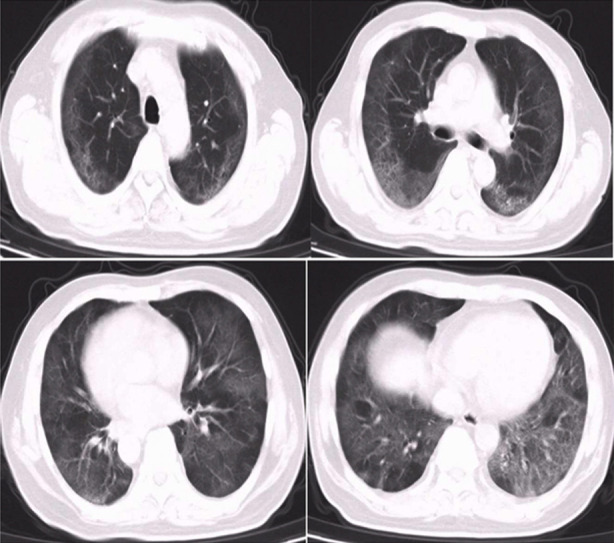
Chest CT scan results of a 53-year-old COVID-19 patient admitted after fever for 4 days. At admission, there were multiple ground-glass changes in both lungs. D-dimer level was 5.56 μg/ml, and hs-CRP level was 22.2 mg/L. He was given antiviral, immunity-enhancing and anticoagulant therapies.

**Fig. 2:**
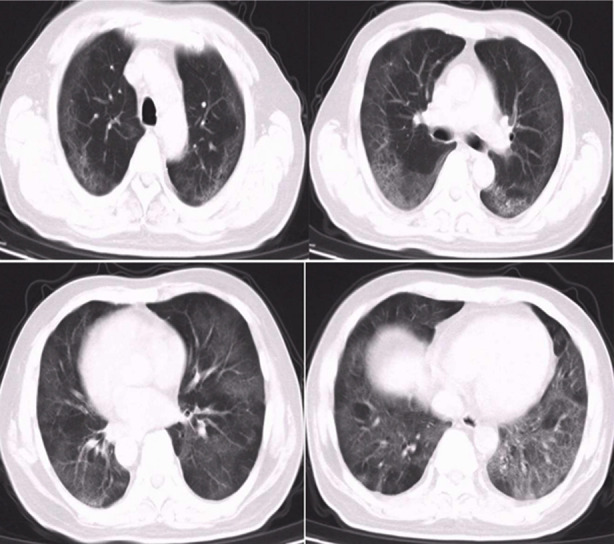
Chest CT scan results after 15 days of hospitalization. Multiple ground-glass lesions were observed, and pulmonary fibrosis was found locally. D-dimer and hs-CRP levels reduced to 0.62 μg/ml and 14.6 mg/L, respectively.

In Group-1, there were two cases of normal chest CT scan results, one case of fibrosis, and 25 cases of abnormalities during the first diagnosis, mainly manifested as single or scattered ground-glass shadows (Fig.1). After treatment, the CT scan results became normal (Fig.2). The chest CT scan of Group-2 showed abnormalities, including 21 cases of multiple ground-glass shadows, and six cases of multiple consolidations accompanied by ground-glass shadows, who were critically ill and died. Additionally, there were 16 cases of multiple ground glass shadows with partial consolidation.

### Laboratory examination results

The hs-CRP levels of most cases significantly increased (53 cases), and those of a few were normal (18 cases). Such levels of 19 cases (26.7%) in Group-1 were elevated, and those of 34 cases (47.8%) in Group-2 were raised. The D-dimer levels of 45 cases were significantly elevated, and those of 26 cases were normal. Only one case was elevated and the rest were normal in Group-1. In Group-2, such levels of 44 cases were raised, accounting for 61.9%.

### Correlation analysis

The levels of hs-CRP and D-dimer in Group-2 significantly exceeded those of Group-1 (Table-I). There were no significant differences in other indices between the two groups (P>0.05). The logistic regression analysis showed that there was a significant positive correlation between D-dimer level and severe symptoms (P<0.05) (Table-II), so this level was an independent risk factor (OR>1) that affected the outcome. The probability of severe outcome rose 1.460-fold (OR: 2.460) with every unit increase of D-dimer level. There were significant positive correlations between chest CT scan results and levels of hs-CRP and D-dimer (r>0, P<0.05).Table-III.

**Table-I:** Baseline clinical data [n(%), (x¯x ± s)].

Index	Group-1 (n=28)	Group-2 (n=43)	χ^2^/t/Z	P
hs-CRP	10.048±8.860	18.236±7.026	-3.059^Δ^	0.002
D-dimer	0.965±0.854	3.273±7.053	-3.765^Δ^	0.000

*: Chi-square test; ^#^: t test; ^Δ^: Z test; ^∇^: Fisher’s exact test.

**Table-II:** Logistic regression analysis results of severe cases.

Item	B	SE	Wald	OR	95%CI		P

					Lower limit	Upper limit	
hs-CRP	0.086	0.044	3.796	1.089	0.999	1.188	0.051
D-dimer	0.900	0.432	4.336	2.460	1.054	5.742	0.037

B: Coefficient estimate; Wald: chi-square value; OR: odds ratio which represents the unit amount of experimental variable increase; CI: confidential interval.

**Table-III:** Correlations of CT scan results with levels of CRP and D-dimer.

Index	r	P
CRP	0.237	0.046
D-dimer	0.288	0.015

## DISCUSSION

COVID-19 belongs to the novel coronavirus of the genus β, with an envelope; the particles are round or oval in shape, which are usually polymorphic, with a diameter of 60 to 140 nm. It is known that the virus is highly contagious, mainly transmitted through respiratory tract and contact. The virus invades the interior of the cell by completing receptor binding with angiotensin-converting enzyme II of mucosal cells.^10^ Serum hs-CRP levels and CT findings of severe patients were consistent with the newly reported pathological manifestations, which were diffuse alveolar lesion under microscope, a large amount of exudative monocytes, lymphocytes and plasma cells in the alveolar cavity and pulmonary interstitium, and extensive pulmonary interstitial fibrosis.^11^ Deng et al. studied the correlation between CT scores and hs-CRP levels at different stages of COVID-19 progression, and obtained a good consistency between CT scores and serum hs-CRP values.^12^ The combination of the two indicators can be used to dynamically and effectively evaluate the changes in the conditions, as well as the efficacy.

D-dimer is one of the specific products of cross-linked fibrin and one of the hallmarks of secondary fibrinolysis *in vivo*. The increased D-dimer content reflects the enhanced activity of secondary fibrinolysis, and the sensitivity of D-dimer in the diagnosis of thrombosis is 95%, so the detection of plasma D-dimer is highly sensitive to the diagnosis of acute thrombosis, which has been widely used in the screening of thrombosis abroad.^13^ Hs-CRP is a cell membrane glycoprotein that stimulates and induces monocytes to express tissue factor which is a key factor that initiates the body’s coagulation waterfall reaction to produce thrombosis, and at the same time, hs-CRP also activates the complement system, and damages vascular intima, thus promoting thrombosis.^14^ The serum hs-CRP levels and CT findings of severe patients were consistent with the newly reported pathological manifestations, which were diffuse alveolar lesion under microscope, a large amount of exudative monocytes, lymphocytes and plasma cells in the alveolar cavity and pulmonary interstitium, and extensive pulmonary interstitial fibrosis.^11^ In this study, 71 patients were divided into mild and severe patients, and the chest CT, hs-CRP, and D-dimer levels were dynamically monitored. In mild patients, early pulmonary lesions are of light density, mainly in the form of cloud and ground glass nodules, with limited scope.^15^ After treatment, pulmonary lesions could be obviously absorbed, even without residual fibrosis. In severe patients, as the disease progressed, the number and density of lesions increased, the scope expanded, and multiple lobes were involved,^16^ and obvious pulmonary fibrosis remained after treatment. In the course of treatment, the hs-CRP and D-dimer levels in severe patients were significantly higher than those in mild patients, showing a progressive increase. After the disease was effectively controlled, most of the young severe patients with good physical fitness had a significantly improved absorption of pulmonary lesions, significantly reduced CT manifestations, decreased density and more common pulmonary fibrosis, the pulmonary lesions of some patients were completely absorbed,^17^ and the levels of hs-CRP and D-dimer fell to the normal range. If the disease continued to progress, it might develop into a critical stage, the CT results showed diffuse consolidation of both lungs, with the manifestations of white lung.^18^ In some patients, the hs-CRP and D-dimer levels continued to rise, the inflammatory response of the body aggravated, complicated with sepsis, leading to systemic multiple organ failure. We found that the hs-CRP and D-dimer levels were significantly increased in severe patients, and the level of increase was consistent with the aggravation degree of chest CT lesions. According to the systemic anatomy of a dead COVID-19 patient, microthrombosis existed in the pulmonary artery.^7^ Therefore, for COVID-19 patients, the D-dimer level should also be closely detected, and routine anticoagulation should be performed when there are no contraindications.

## CONCLUSION

Many studies have reported that chest CT progress is positively correlated with elevated hs-CRP levels, which was also found in this study. However, the hs-CRP level is closely related to the occurrence of thrombosis, and D-dimer levels in critical patients continue to increase. Chest CT scan, hs-CRP and D-dimer levels have good consistency. In the clinical process of diagnosis and treatment, if the scope of chest CT lesions is expanded and the disease progresses rapidly, hs-CRP and D-dimer levels can be closely monitored. If anticoagulant therapy is given without contraindications, the conditions of most patients can be significantly alleviated, and dynamic and effective evaluation of the condition changes can be performed in combination with several indicators, and the therapeutic effect can also be evaluated, so as to help timely adjust the diagnosis and treatment plan in clinical practice.

### Authors’ Contributions:

**JZ & BL:** Study design and significant manuscript revision.

**CC & RS:** manuscript drafting, clinical data collection and analysis.

**JZ, CC, RS & BL:** approval of manuscript submission.
